# Association of Red Meat Intake with the Risk of Cardiovascular Mortality in General Japanese Stratified by Kidney Function: NIPPON DATA80

**DOI:** 10.3390/nu12123707

**Published:** 2020-11-30

**Authors:** Hiroyoshi Segawa, Keiko Kondo, Aya Kadota, Hiromi Yamauchi, Seiko Ohno, Sachiko Tanaka-Mizuno, Nagako Okuda, Naoko Miyagawa, Hisatomi Arima, Tomonori Okamura, Katsuyuki Miura, Akira Okayama, Hirotsugu Ueshima, the NIPPON DATA80 Research Group

**Affiliations:** 1 Center for Epidemiologic Research in Asia, Shiga University of Medical Science, Seta Tsukinowa-cho, Otsu 520-2192, Japan; ayakd@belle.shiga-med.ac.jp (A.K.); hiromiy@belle.shiga-med.ac.jp (H.Y.); miura@belle.shiga-med.ac.jp (K.M.); hueshima@belle.shiga-med.ac.jp (H.U.); 2 Department of Public Health, Shiga University of Medical Science, Seta Tsukinowa-cho, Otsu 520-2192, Japan; kon@belle.shiga-med.ac.jp; 3 Department of Bioscience and Genetics, National Cerebral and Cardiovascular Center, 6-1 Kishibe-Shimmachi, Suita 564-8565, Japan; sohno@ncvc.go.jp; 4 Department of Medical Statistics, Shiga University of Medical Science, Seta Tsukinowa-cho, Otsu 520-2192, Japan; sachikot@belle.shiga-med.ac.jp; 5 Department of Health and Nutrition, University of Human Arts and Sciences, 1288 Magome, Iwatsuki-ku, Saitama 399-8539, Japan; nagako_okuda@human.ac.jp; 6 International Center for Nutrition and Information, National Institutes of Biomedical Innovation, Health and Nutrition, 1-23-1 Toyama, Shinjuku-ku, Tokyo 162-8636, Japan; naocom@belle.shiga-med.ac.jp; 7 Department of Preventive Medicine and Public Health, Faculty of Medicine, Fukuoka University, 7-45-1 Nanakuma, Jonan-ku, Fukuoka 814-0180, Japan; harima@fukuoka-u.ac.jp; 8 Department of Preventive Medicine and Public Health, Keio University School of Medicine, 35 Shinanomachi, Shinjuku-ku, Tokyo 160-8582, Japan; okamura@z6.keio.jp; 9 Research Institute of Strategy for Prevention, Miyazaki Shinkawa Building 4F, 1-3-9 Shinkawa, Chuo-ku, Tokyo 104-0033, Japan; aokayama@jrisp.com

**Keywords:** red meat, kidney function, cardiovascular mortality

## Abstract

The consumption of red meat has been recommended for individuals with reduced kidney function. However, red meat intake was recently suspected to increase cardiovascular disease (CVD) risk. We evaluated the association of red meat intake with CVD mortality risk in Japanese with/without reduced kidney function. Overall, 9112 participants of a Japanese national survey in 1980, aged ≥30 years, were followed for 29 years. Red meat intake was assessed using weighed dietary record. Cox proportional hazards models were used to estimate the hazard ratio (HR) of CVD mortality according to sex-specific tertiles of red meat intake. We also performed stratified analyses with/without reduced kidney function defined as estimated glomerular filtration rate less than 60 mL/min/1.73 m^2^. Red meat intake was not associated with CVD mortality risk in men and women. In stratified analyses, the HR of the highest compared with the lowest tertile of red meat intake was lower only in women with reduced kidney function (0.67, 95% confidence interval 0.46–0.98). In conclusion, there were no clear associations between red meat intake and CVD mortality risk in Japanese population; however, a higher intake of red meat was associated with lower risk of future CVD mortality in women with reduced kidney function.

## 1. Introduction

Reduced kidney function is currently recognized as a risk factor for cardiovascular disease (CVD) [1,2]. To prevent kidney failure, people with reduced kidney function have been advised to restrict protein intake and choose a protein source of high biological value, including red meat [3], to avoid essential amino acid deficiency [4,5]. However, red meats such as beef, pork, and lamb, which is a major origin of saturated fatty acid, were shown to be positively associated with the risk of CVD [6,7,8]. As a result of this conflicting advice, some researchers have raised concerns about patients with reduced kidney function consuming too much red meat [9]. However, to date, there have been no reports showing an association between red meat intake and CVD risk among people with reduced kidney function.

The aim of this study was to evaluate the impact of red meat intake on CVD mortality risk in people with and without reduced kidney function in a long-term cohort study of the Japanese general population.

## 2. Materials and Methods

### 2.1. Study Participants

The National Integrated Project for Prospective Observation of Non-communicable Disease And its Trends in the Aged (NIPPON DATA80) is a prospective, nationwide, population-based cohort study involving men and women aged 30 years and older who participated in the National Survey on Circulatory Disorders of Japan and the National Nutrition Survey of Japan (NNSJ) in 1980 [10,11,12]. From 300 randomly selected survey districts, 10,546 people (4639 men and 5907 women) agreed to participate, with a participation rate of 76.6%. All procedures performed in studies were in accordance with the ethical standards of the institutional and/or national research committee at which the studies were conducted (IRB approval number R2005-021) and with the 1964 Helsinki declaration and its later amendments or comparable ethical standards.

In the present study, we excluded 1434 people for the following reasons: absence of a present address, which was needed to link the participants to their vital statistics records (*n* = 909), missing nutritional data (*n* = 87), missing baseline characteristics (*n* = 171), history of CVD (*n* = 248), total energy intake >5000 or <500 kcal/day (*n* = 14), and estimated glomerular filtration rate (eGFR) <15 mL/min/1.73 m^2^ (*n* = 5). The remaining 9112 participants (3986 men and 5126 women) were included in the analysis.

### 2.2. Baseline Survey

Baseline characteristics were obtained by physical examinations, blood tests, and a series of questionnaires. Drinking and smoking habits were reported by participants according to “every day, sometimes, never, or ex-drinker” and “current, never, ex-smoker”, respectively. Trained observers measured blood pressure in each participant with a standard mercury sphygmomanometer after a 5-min rest period. Casual blood and urine samples obtained in each health center were collected and analyzed in a specific laboratory (Center for Adult Diseases, Osaka, Japan). Proteinuria was defined as proteinuria positive or more with a urine dipstick test. Serum glucose, total cholesterol, albumin, and uric acid were analyzed by an autoanalyzer (SMA12/60; Technicon, Tarrytown, NY, USA). Serum glucose was measured by the cupric-neocuproine method using an autoanalyzer. Since more recent measurements of blood glucose levels use the hexokinase method, serum glucose levels were adjusted using the formula (0.047 × (glucose concentration in mg/dL) − 0.541) (mmol/L) [13,14]. In the present study, diabetes mellitus was defined as serum glucose levels of 11.1 mmol/L or higher, history of diabetes, or both. Serum creatinine levels were measured with the alkaline picric acid (Jaffe) method. Since the eGFR can be derived by creatinine measured using an enzymic method, serum creatinine levels were transformed to that of the enzyme method by subtracting 0.2 from the measured creatinine level (mg/dL). Then, eGFR was calculated for each participant using the following equation: eGFR (mL/min/1.73 m^2^) = 194 × serum creatinine (mg/dL)^−1.094^ × age^−1.287^ × 0.739 (if women) [15,16]. Reduced kidney function was defined as an eGFR lower than 60 mL/min/1.73 m^2^.

### 2.3. Nutritional Survey

In the NNSJ in 1980, food consumption data were collected for 3 consecutive days, excluding Saturday, Sunday, and national holidays. Participants were asked to weigh and record all food and beverages consumed by household members and report the amounts. The accuracy of food intake data was assessed and confirmed by well-trained dietitians. Dietary records were coded using the Standard Tables for Foods in Japan (3rd edition), and the intake of each food group was calculated for every household. Detailed methods are described elsewhere [17,18].

Red meat intake was calculated as the total amount of pork, beef, ham, and sausage consumed. Means and standard deviations were derived for food group intake (per 1000 kcal), nutrient intake (per 1000 kcal), and nutrient densities of protein, fat, and carbohydrate (%kcal). All food groups and nutrient intakes were calculated as a simple density measurement: total intake of household divided by the household energy intake. Total energy (kcal/day) was calculated by distributing intakes in each household considering the age and sex of individuals [11].

### 2.4. Follow-up Survey

In the present study, the participants were followed for 29 years until 2009 (1980–2009). We used vital statistics data to identify the cause of death with the permission of the Ministry of Health, Labor and Welfare, Japan. The procedure used for endpoint determination was reported elsewhere [12]. The underlying causes of death were assigned centrally according to the International Classification of Diseases, Ninth Revision (ICD-9), through to the end of 1994 and according to the International Classification of Diseases, Tenth Revision (ICD-10), from the beginning of 1995. Death from total CVD (ICD-9: 393–459 and ICD-10: 100–199) was the primary endpoint in this study. Participants who were lost during follow-up were censored at the date of last follow-up.

### 2.5. Statistical Analysis

A Cox proportional hazards model was used to evaluate the risk of CVD mortality for higher red meat intake. First, the participants were stratified into tertiles of red meat intake regardless of kidney function, and hazard ratios (HRs) of CVD mortality compared with the lowest-intake group were calculated. Next, we stratified participants according to the presence of reduced kidney function (eGFR ≥ 60 or eGFR < 60) and calculated HRs for CVD mortality. We also assessed the statistical interaction between red meat intake and kidney function (eGFR ≥ 60 or eGFR < 60 mL/min/1.73 m^2^) for CVD mortality risk.

All analyses were performed separately by sex. We calculated HRs in an age-adjusted model and a multivariable adjusted model. The multivariable adjusted model was adjusted for age, body mass index, smoking status (current, never, or ex-smoker), drinking status (every day, sometimes, never, or ex-drinker), diabetes mellitus (yes or no), systolic blood pressure, proteinuria (yes or no), and intakes of vegetables, fruit, and salt. All variables except smoking status, drinking status, diabetes mellitus, and proteinuria were included as continuous variables in the model. In addition, we used restricted cubic splines with 3 knots to evaluate a dose–response association of red meat intake with CVD mortality.

All analyses were performed using SAS version 9.4 (SAS Institute, Cary, NC, USA). All statistical tests were two sided, and *p* < 0.05 was considered statistically significant.

## 3. Results

### 3.1. Baseline Characteristics of Study Participants

Baseline characteristics of study participants are shown in Table 1. The mean age of men and women was 50.3 and 50.7 years, respectively. The mean total energy intake (kcal/day) was higher in men than in women. The density of red meat intake (g/1000 kcal) was similar between men and women. The proportion of participants with reduced kidney function was 11.2% for men and 12.9% for women. Baseline characteristics of participants according to tertiles of red meat intake (Table S1) and those stratified by kidney function (Table S2) are shown in supplementary materials.

### 3.2. Red Meat Intake and CVD Mortality Risk

During 29 years and 221,357 person-years of follow-up, there were 1117 deaths (532 men and 585 women) attributable to CVD. Table 2 shows the hazard ratios of CVD mortality according to tertiles of red meat intake among overall participants. Red meat intake was not significantly associated with the risk of CVD mortality in men (*p* for trend = 0.471) and women (*p* for trend = 0.915).

Table 3 shows the hazard ratios of CVD mortality according to tertiles of red meat intake stratified by kidney function. In men, no significant association between red meat and CVD mortality was observed regardless of kidney function (*p* for interaction = 0.266). In women without reduced kidney function, the CVD mortality risk tended to be higher in the highest tertile of red meat intake compared with the lowest tertile, although this did not reach statistical significance (HR 1.22 [95% confidence interval (CI), 0.95–1.56]). By contrast, the CVD mortality risk was significantly lower in the highest tertile of red meat intake compared with the lowest tertile of red meat intake in women with reduced kidney function (HR 0.67 [95% CI, 0.46–0.98]). This indicated a significant interaction between red meat intake and the presence of reduced kidney function in women (*p* = 0.012).

In spline regression analysis, red meat intake was not associated with the risk of CVD mortality in men and women. Red meat intake tended to inversely associate with CVD mortality in women with reduced kidney function (Figures S1 and S2).

## 4. Discussion

In this population-based prospective cohort study of the Japanese general population, we assessed the long-term CVD mortality risk for higher red meat intake in people with and without reduced kidney function. Red meat intake was not significantly associated with the risk of CVD mortality in men and women. In the stratified analysis by kidney function, the association of red meat intake with CVD mortality was not observed in men. However, higher red meat intake was associated with a lower risk of CVD mortality in women with reduced kidney function. To the best of our knowledge, this is the first study to show the association between red meat intake and CVD mortality by the stratified analysis of kidney function.

Among animal proteins, red meat including beef and pork is a major protein source alongside fish in Japan. However, red meat is also the major origin of saturated fatty acid, which increases serum low-density lipoprotein levels [19], and if consumed in high amounts, it is associated with a high risk of CVD [6]. Although some reports have shown a positive association between red meat intake and CVD risk in Western countries [7,8], Japanese and Asian cohort studies have failed to show such a relationship [20,21]. In the present study, red meat intake was not positively associated with CVD mortality, which is in line with previous Asian studies. We considered salt intake and subsequent high blood pressure as other residual confounding factors, neither of which was adjusted for in previous Japanese/Asian reports. Historically, before the development of refrigerators or freezers, fish, unlike meat, was often eaten as a food preserved by the addition of salt. Therefore, people who prefer red meat to fish might consume relatively less salt. Indeed, in the NIPPON DATA80 cohort, meat intake was inversely associated with salt intake, while fish intake was positively associated with salt intake [22]. However, a positive association was not observed even after the adjustment for salt intake and systolic blood pressure in the present study. Japanese/Asian people consume a small amount of red meat compared with their Western counterparts [23]. In addition, the proportion of processed red meat intake is higher in Western people than in Asian people [24] as well as our study participants. Since processed meat is usually high in salt, a positive association between red meat intake and CVD in Western people might be confounded by salt. Those might explain this inconsistency.

We observed an inverse association between red meat intake and risk of CVD mortality in women with reduced kidney function. Although the mechanisms involved are unclear, various nutrients might partly explain this association. A study by Nagao et al. reported that animal foods rich in branched-chain amino acids, including L-arginine, tryptophan, and tyrosine, protected the heart from ischemic damage [20]. Furthermore, red meat is an important origin of bioactive compounds (e.g., l-carnitine and coenzyme Q10), which help energy generation, reduce oxidative stress, and might protect against CVD [25]. Therefore, a modest intake of red meat by women with reduced kidney function who are at high risk of CVD might protect from CVD.

However, a significant association between red meat and CVD mortality was not observed in men regardless of kidney function. We considered several reasons for the sex differences. First, the absolute amount of red meat intake was lower in women than in men, although the simple density value was similar. Therefore, red meat might have protective effects for CVD among women consuming low amounts of red meat. Second, the prevalence of smoking and alcohol drinking was much higher in men than in women. Although these competing risk factors were statistically adjusted, remaining confounding factors might have attenuated the association between red meat intake and CVD mortality risk in men. Third, the inverse association was shown only in women with reduced kidney function in our stratified analyses. This significant association in only one subgroup might be a chance finding. Fourth, the subgroup analyses might not have had sufficient power of detection.

Our findings were based on a dietary survey conducted in 1980. Japanese dietary habits have become Westernized over time, and red meat intake has increased from 24.2 g/1000 kcal in 1980 to 35.3 g/1000 kcal in 2017 [26]. Therefore, we should carefully extrapolate the result to the present situation. Further studies are needed to clarify the optimal intake of red meat for people with reduced kidney function.

This study had several limitations. First, there was a significant age difference between participants with and without reduced kidney function. Although we adjusted for age when performing the analyses, the influence of age could not be ignored. Second, data on food and nutrient intake were collected only at baseline. Participants might have changed their diet pattern during the follow-up period. Moreover, dietary records from 3 consecutive days are influenced by seasonal dietary habit. This dietary assessment might not reflect the participants’ representative dietary habits. Third, participants’ red meat consumption was assumed on the basis of the household intake and thereby might not reflect an individual’s consumption of red meat. Fourth, the serum creatinine level was measured only once at baseline, which might lead to a misclassification of kidney function. Furthermore, we could not take into account the change of dietary recommendation from kidney disease with preserved kidney function to end stage kidney disease, which needs renal replacement therapy [27,28]. Fifth, the leading cause of chronic kidney disease (CKD) in Japan in the 1980s was chronic glomerular nephritis [29]. The prevalence of diabetes mellitus was much lower in the present study than that reported more recently [30]. As a result of the difference in the etiology of reduced kidney function, the present results may not be compatible with a more contemporary population. Finally, we could not obtain the data of social factors. It is possible that social factors such as income might affect the association between red meat intake and CVD mortality risk.

## 5. Conclusions

In the present study, there were no clear associations between red meat intake and CVD mortality risk in Japanese population; however, a higher intake of red meat was associated with lower risk of future CVD mortality in women with reduced kidney function. Further investigation is required to confirm the effect of red meat intake on CVD risk.

## Figures and Tables

**Table 1:** Baseline characteristics of participants (3986 men and 5126 women, aged ≥30 years, NIPPON DATA80, 1980).

	Total (*n* = 9112)	Men (*n* = 3986)	Women (*n* = 5126)
Age (years)	50.5 ± 13.2	50.3 ± 13.1	50.7 ± 13.3
BMI (kg/m^2^)	22.7 ± 3.2	22.5 ± 2.9	22.9 ± 3.4
Estimated GFR (mL/min/1.73 m^2^)	79.4 ± 18.2	78.6 ± 16.4	80.0 ± 19.5
Reduced renal function ^†^ (*n* [%])	1104 (12.1)	445 (11.2)	659 (12.9)
Systolic blood pressure (mmHg)	135.8 ± 21.3	138.3 ± 20.8	133.9 ± 21.4
Diastolic blood pressure (mmHg)	81.3 ± 12.2	83.6 ± 12.3	79.6 ± 11.8
Creatinine (μmol/L) ^#^	65.0 ± 15.8	75.5 ± 13.9	56.9 ± 11.8
Total cholesterol (mmol/L)	4.9 ± 0.9	4.8 ± 0.8	4.9 ± 0.9
Albumin (g/L)	43.9 ± 2.7	44.3 ± 2.9	43.6 ± 2.5
Uric acid (μmol/L)	295.6 ± 78.5	342.2 ± 74.8	259.4 ± 60.0
Casual blood glucose (mmol/L)	5.6 ± 1.6	5.6 ± 1.7	5.5 ± 1.6
Nutrients and foods intake *			
Total energy (kcal/day)	2139.9 ± 490.4	2405.9 ± 478.3	1933.0 ± 389.7
Protein (% kcal)	15.1 ± 2.1	15.1 ± 2.1	15.1 ± 2.0
Fat (% kcal)	21.6 ± 5.5	21.5 ± 5.4	21.7 ± 5.5
Carbohydrate (% kcal)	60.8 ± 6.2	60.7 ± 6.2	60.8 ± 6.1
Salt (g/1000 kcal)	6.3 ± 2.1	6.3 ± 2.1	6.3 ± 2.1
Vegetables (g/1000 kcal)	121.6 ± 45.1	120.6 ± 44.5	122.5 ± 45.5
Fruit (g/1000 kcal)	76.2 ± 46.5	74.3 ± 45.8	77.7 ± 47.0
Red meat (g/1000 kcal)	22.0 ± 14.1	22.2 ± 14.2	21.9 ± 14.1
Unprocessed red meat (g/1000 kcal)	18.2 ± 12.7	18.3 ± 12.8	18.1 ± 12.7
Processed meat (g/1000 kcal)	3.8 ± 4.8	3.8 ± 4.8	3.8 ± 4.7
Smoking			
Current (*n* [%])	2970 (32.6)	2521 (63.3)	449 (8.8)
Never (*n* [%])	5310 (58.3)	742 (18.6)	4568 (89.1)
Ex-smoker (*n* [%])	832 (9.1)	723 (18.1)	109 (2.1)
Drinking			
Every day (*n* [%])	2068 (22.7)	1923 (48.2)	145 (2.8)
Sometimes (*n* [%])	1937 (21.3)	1059 (26.6)	878 (17.1)
Never (*n* [%])	4822 (52.9)	795 (19.9)	4027 (78.6)
Ex-drinker (*n* [%])	285 (3.1)	209 (5.2)	76 (1.5)
Proteinuria (*n* [%])	231 (2.5)	116 (2.9)	115 (2.2)
Diabetes mellitus (*n* [%])	339 (3.7)	206 (5.2)	133 (2.6)
Antihypertensive medication (*n* [%])	959 (10.5)	384 (9.6)	575 (11.2)

GFR, glomerular filtration rate. Continuous variables are described as the mean ± standard deviation. ^†^ Reduced renal function was defined as an eGFR < 60 mL/min/1.73 m^2^. ^#^ Creatinine was adjusted to enzymic method values. * Data of nutrients and food intake are described as household simple density except total energy.

**Table 2:** Hazard ratios of cardiovascular disease (CVD) mortality according to tertiles of red meat intake.

	Tertiles of Red Meat Intake (g/1000 kcal)			*p* for Trend
	Tertile 1	Tertile 2	Tertile 3	
		HR (95% CI)	HR (95% CI)	
Men				
Range of red meat intake (g/1000 kcal)	<14.9	15.0–26.4	>26.4	
Mean of red meat intake (g/day)	20.1 ± 12.3	49.1 ± 12.2	91.1 ± 29.1	
No. of participants	1332	1326	1328	
No. of CVD death	213	148	171	
Age adjusted model	1	0.84 (0.68–1.04)	0.98 (0.80–1.20)	0.897
Multivariable adjusted model	1	0.90 (0.73–1.11)	1.08 (0.88–1.33)	0.471
Women				
Range of red meat intake (g/1000 kcal)	<14.9	14.9–26.3	>26.3	
Mean of red meat intake (g/day)	15.2 ± 9.8	39.4 ± 10.0	73.4 ± 24.3	
No. of participants	1709	1706	1711	
No. of CVD death	249	176	160	
Age adjusted model	1	0.94 (0.77–1.14)	0.99 (0.81–1.20)	0.870
Multivariable adjusted model	1	0.96 (0.79–1.17)	1.01 (0.83–1.24)	0.915

Multivariable adjusted model was adjusted for age, body mass index, smoking (current, never, or ex-smoker), drinking (every day, sometimes, never, or ex-drinker), diabetes mellitus, systolic blood pressure, proteinuria, vegetables, fruit, and salt intake. Red meat intake (g/day) is described as the mean ± standard deviation. CVD, cardiovascular disease; HR, hazard ratio; CI, confidence interval.

**Table 3:** Hazard ratios of CVD mortality according to tertiles of red meat intake stratified by kidney function.

	Tertiles of Red Meat Intake (g/1000 kcal)			*p* for Trend	*p* for Interaction *
	Tertile 1	Tertile 2	Tertile 3		
		HR (95% CI)	HR (95% CI)		
Men					
eGFR ≥ 60					
No. of participants	1165	1182	1194		
No. of CVD death	161	106	121		
Age adjusted model	1	0.80 (0.62–1.02)	0.92 (0.73–1.17)	0.509	
Multivariable adjusted model	1	0.83 (0.65–1.06)	1.03 (0.81–1.31)	0.823	
eGFR < 60					
No. of participants	167	144	134		
No. of CVD death	52	42	50		
Age adjusted model	1	0.94 (0.63–1.41)	1.11 (0.75–1.64)	0.580	
Multivariable adjusted model	1	1.00 (0.65–1.52)	1.05 (0.69–1.60)	0.825	0.266
Women					
eGFR ≥ 60					
No. of participants	1461	1477	1529		
No. of CVD death	162	118	116		
Age adjusted model	1	0.93 (0.73–1.18)	1.16 (0.91–1.47)	0.251	
Multivariable adjusted model	1	0.97 (0.76–1.24)	1.22 (0.95–1.56)	0.134	
eGFR < 60					
No. of participants	248	229	182		
No. of CVD death	87	58	44		
Age adjusted model	1	0.96 (0.68–1.34)	0.69 (0.48–0.99)	0.047	
Multivariable adjusted model	1	0.91 (0.64–1.29)	0.67 (0.46–0.98)	0.039	0.012

Multivariable adjusted model was adjusted for age, body mass index, smoking (current, never, or ex-smoker), drinking (every day, sometimes, never, or ex-drinker), diabetes mellitus, systolic blood pressure, proteinuria, vegetables, fruit and salt intake. CVD, cardiovascular disease; HR, hazard ratio; CI, confidence interval. * Multivariable adjusted interaction term between red meat intake and kidney function (dichotomous; eGFR ≥ 60, <60 mL/min/1.73 m^2^).

## Supplementary Materials

The following are available online at https://www.mdpi.com/2072-6643/12/12/3707/s1, Table S1: Baseline characteristics of participants according to tertiles of red meat intake (3986 men and 5126 women, aged ≥30 years, NIPPON DATA80, 1980). Table S2: Baseline characteristics of participants by kidney function (3986 men and 5126 women, aged ≥30 years, NIPPON DATA80, 1980). Figure S1: Multivariable-adjusted relationship of red meat intake with CVD mortality, evaluated using restricted cubic splines in men. Multivariable adjusted model was adjusted for age, body mass index, smoking (current, never, or ex-smoker), drinking (every day, sometimes, never, or ex-drinker), diabetes mellitus, systolic blood pressure, proteinuria, vegetables, fruit and salt intake. We used restricted cubic splines with 3 knots; Figure S2: Multivariable-adjusted relationship of red meat intake with CVD mortality, evaluated using restricted cubic splines in women. Multivariable adjusted model was adjusted for age, body mass index, smoking (current, never, or ex-smoker), drinking (every day, sometimes, never, or ex-drinker), diabetes mellitus, systolic blood pressure, proteinuria, vegetables, fruit and salt intake. We used restricted cubic splines with 3 knots.
