# Supplementary material for: Association of Red Meat Intake with the Risk of Cardiovascular Mortality in General Japanese Stratified by Kidney Function: NIPPON DATA80
Source: Nutrients. 2020 Nov 30;12(12):3707. doi: 10.3390/nu12123707 (PMC7761136; doi:10.3390/nu12123707)
Supplement: Supplementary file 1 [file nutrients-12-03707-s001.zip › nutrients-1018407-suppl/nutrients-1018407-suppl figure.pdf]

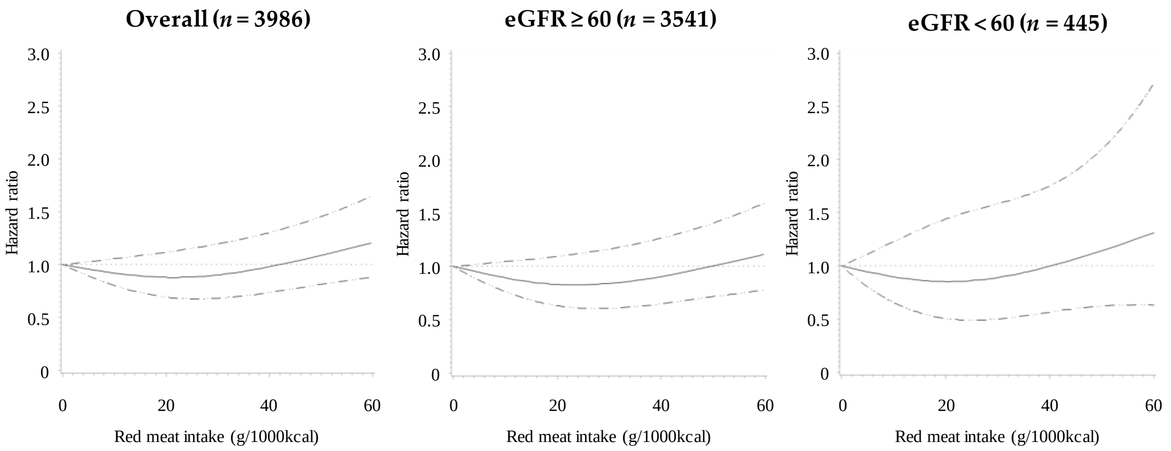

Figure S1. Multivariable-adjusted relationship of red meat intake with CVD mortality, evaluated using restricted cubic splines in men. Multivariable adjusted model was adjusted for age, body mass index, smoking (current, never, or ex-smoker), drinking (every day, sometimes, never, or ex-drinker), diabetes mellitus, systolic blood pressure, proteinuria, vegetables, fruit and salt intake. We used restricted cubic splines with 3 knots.

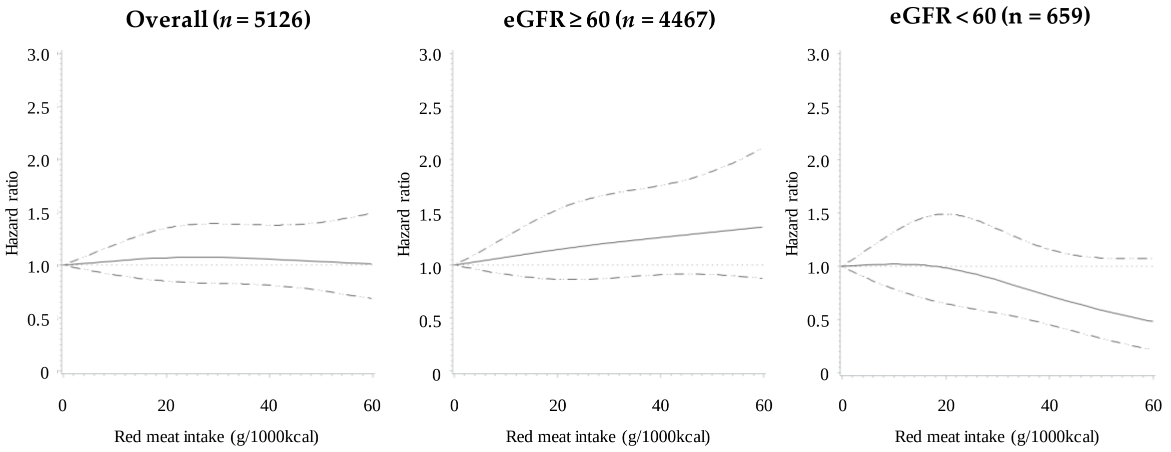

Figure S2. Multivariable-adjusted relationship of red meat intake with CVD mortality, evaluated using restricted cubic splines in women. Multivariable adjusted model was adjusted for age, body mass index, smoking (current, never, or ex-smoker), drinking (every day, sometimes, never, or ex-drinker), diabetes mellitus, systolic blood pressure, proteinuria, vegetables, fruit and salt intake. We used restricted cubic splines with 3 knots.
